# Neuropathology of central nervous system involvement in TTR amyloidosis

**DOI:** 10.1007/s00401-022-02501-9

**Published:** 2022-10-06

**Authors:** Ricardo Taipa, Luísa Sousa, Miguel Pinto, Inês Reis, Aurora Rodrigues, Pedro Oliveira, Manuel Melo-Pires, Teresa Coelho

**Affiliations:** 1 Portuguese Brain Bank, Neuropathology Unit, Department of Neurosciences, Centro Hospitalar Universitário do Porto, Largo Prof. Abel Salazar, 4099-001 Porto, Portugal; 2 UMIB, Unit for Multidisciplinary Research in Biomedicine, ICBAS, School of Medicine and Biomedical Sciences, University of Porto, Porto, Portugal; 3 Laboratory for Integrative and Translational Research in Population Health, ITR, Porto, Portugal; 4 Department of Neurology, Centro Hospitalar de Entre o Douro e Vouga, Santa Maria da Feira, Portugal; 5 Epidemiological Research Unit (EPIUnit), ICBAS, School of Medicine and Biomedical Sciences, University of Porto, Porto, Portugal; 6 Unidade Corino de Andrade, Department of Neurosciences, Centro Hospitalar Universitário do Porto, Porto, Portugal

**Keywords:** Transthyretin, Cerebral amyloid angiopathy, Neuropathology, Stages, Central nervous system

## Abstract

Hereditary transthyretin amyloidosis (ATTRv) is a systemic disease caused by the accumulation of misfolded transthyretin (TTR). It usually presents with an adult-onset progressive axonal peripheral neuropathy and cardiomyopathy. In the central nervous system (CNS), variant TTR is produced by the choroid plexus and accumulates in the leptomeninges. CNS symptoms have been increasingly recognized in this population, including transient focal neurological episodes and stroke, particularly in patients with the V30M mutation and longstanding disease. The prevalence, pathophysiology, and progression of CNS involvement remain to be clarified. The present work explores if there is a recognizable sequence of CNS TTR deposition in ATTRv. We studied the topographical and severity distribution of TTR deposition in 16 patients with ATTRv, aged 27–69 years and with a mean disease duration of 10.9 years (range: 3–29). Our results suggest that CNS pathological involvement in V30M ATTRv occurs early in the disease course, probably starting in pre-symptomatic phases, and follows a distinct sequence. Leptomeninges and subarachnoid meningeal vessels are affected earlier, then followed by perforating cortical vessels and subpial deposition, and finally by deposition in the subependymal and basal ganglia vessels near the ependymal lining. Brainstem and spinal cord show early and severe involvement, with amyloid subpial deposition already seen in initial stages. Despite massive superficial amyloid deposition, no parenchymal deposition outside subpial or subependymal regions was found. Additionally, vascular lesions or superficial cortical siderosis were not frequent. Future studies with more patients from different populations and TTR mutations will be important to confirm these findings. Defining stages of TTR pathology in the CNS may be useful to better understand pathogenic mechanisms leading to symptoms and to interpret neuroimaging biomarkers.

**Supplementary Information:**

The online version contains supplementary material available at 10.1007/s00401-022-02501-9.

## Introduction

Hereditary transthyretin amyloidosis (ATTRv amyloidosis; v for variant) is a genetic disease caused by the accumulation of misfolded transthyretin protein in different organs [1, 11]. The most common mutation is V30M (p.Val50Met), but more than 130 mutations are recognized producing a broad range of phenotypes [1, 22]. Clinical manifestations include an adult-onset progressive axonal peripheral neuropathy, cardiomyopathy, and variable renal and ocular involvement [1, 2, 12].

Central nervous system (CNS) manifestations also seem common, particularly in patients with the V30M mutation and longstanding disease [21, 27]. The most common CNS manifestations are transient focal neurological episodes (TFNEs), in which patients show short, self-limited, stereotyped episodes of focal cortical dysfunction. TFNEs are found in 12–31% of patients in post-transplant cohorts, usually after 14 years of disease duration [21, 27]. Less frequently, hemorrhagic and ischemic stroke are also described [21, 33]. The pathophysiology of CNS symptoms remains to be clarified. Transthyretin production by the choroid plexus escapes the therapeutic effect of liver transplant and other approved disease modifying therapies, which do not cross the blood–brain barrier. This allows for the continuous accumulation of amyloid in the CNS throughout the disease [30].

Despite being a marginal issue in research, CNS pathologic involvement was described early after the first description of the disease [2, 13]. The few published studies show a universal amyloid deposition in leptomeningeal vessels and pia-arachnoid membranes, causing a cerebral amyloid angiopathy (CAA) and sparing the parenchyma [32]. It is still unknown how TTR deposition progresses throughout the disease course and what is the frequency and type of vascular lesions in these patients.

The present work explores if there is a recognizable sequence of CNS TTR deposition in ATTRv. Our findings suggest a distinct sequence of CNS involvement, with leptomeninges and subarachnoid vessels affected earlier, followed by perforating cortical vessels and subpial deposition, and finally subependymal deposition and involvement of basal ganglia vessels near the ependymal lining. Interestingly, brainstem and spinal cord show early severe involvement, with amyloid subpial deposition already seen in the initial stages. Despite massive CNS amyloid deposition, no parenchymal deposition was found in the cortex or white matter.

These findings are important to understand CNS pathological involvement of ATTRv amyloidosis, particularly regarding amyloid-neuroimaging distribution patterns.

## Materials and methods

### Clinical data

Sixteen human autopsy brains of patients with ATTRv, aged 27–69 years, were studied. The cases were identified from the archive of Unidade de Neuropatologia of Centro Hospitalar Universitário do Porto (CHUP) (case #1 to #12) and from the Portuguese Brain Bank (PBB) (cases #13 to #16). Seven cases (#1 to #3 and #6 to #9) were previously described [21]. All cases were followed in the Neurology outpatient clinic of CHUP, and clinical data were retrospectively assessed. Genetic testing in cases #1 to #3 was not performed, because at that time, no molecular diagnosis was available. Appropriate consent procedures for the collection and use of human brain tissues were obtained and the study was approved by the Ethics Committee of CHUP.

### Neuropathological analysis

Tissue was fixed in a 4% aqueous solution of formaldehyde. Paraffin sections (4 µm) were obtained from the following anatomical regions: frontal cortex, entorhinal region, cerebellum, thalamus, basal ganglia, midbrain, pons, medulla, and upper cervical cord. Cases #1, #5, #8, and #11 did not have cervical cord section. When available, particularly from the PBB cases, additional regions were included: temporal, parietal and occipital cortices, cingulate cortex, amygdala region, and olfactory bulb.

All cases were studied for the presence of neurodegenerative disorders and vascular pathology. Cortical cerebral microinfarcts were defined as microscopic regions of cellular death or tissue necrosis up to 5 mm in dimension and lacunar infarcts as cavitating infarcts measuring up to 15 mm [14]. Territorial infarcts and hemorrhages were also recorded. Microhemorrhages were distinguished from perivascular haemosiderin leakage by the accumulation of haemosiderin in the brain parenchyma [28].

### Histology and immunohistochemistry

Six μm-thick paraffin-embedded tissue sections from the representative blocks were cut on a microtome. Haematoxylin & Eosin, Perls’ Prussian blue, and Congo red staining were performed using standard protocols. For the Perls’ protocol sections were incubated with a 1:1 mixture of 5% hydrochloric acid and 5% potassium ferrocyanide (30 min), and counterstained with eosin (1 min). For the Congo red, protocol sections were place in Congo Red solution (8 min), followed by differentiation into 0.2% alcoholic Potassium Hydroxide (quickly), 0.2% aqueous Potassium Hydroxide (quickly), washed in distilled water, and counterstained with hematoxylin (less than 1 min).

Immunohistochemistry (IHC) staining was performed using the Ventana OptiView DAB IHC detection kit and the Ventana BenchMark Ultra processor (Ventana, Tucson, AZ, USA). Paraffin tissue sections after dewax were pre-treated with heat using Ultra Cell Conditioning Solution (CC1 or CC2; Ventana) and the endogenous peroxidase was inactivated before the incubation with the primary antibodies: α-synuclein (Novocastra, KM51, 1/80), β-amyloid (Dako, 6F/3D, 1/120, Tau (Thermoscientific, AT8, 1:1200), TDP-43 (Proteintech, ref: 10,782–2-AP, 1/2000), or TTR (Dako, ref: A 0002, 1/1500). The slides immunostained with α-synuclein and β-amyloid antibodies suffered an extra pre-treatment with formic acid for 2 and 1 min, respectively, before the heat pre-treatment.

### Morphological analysis

**Fig. 1:**
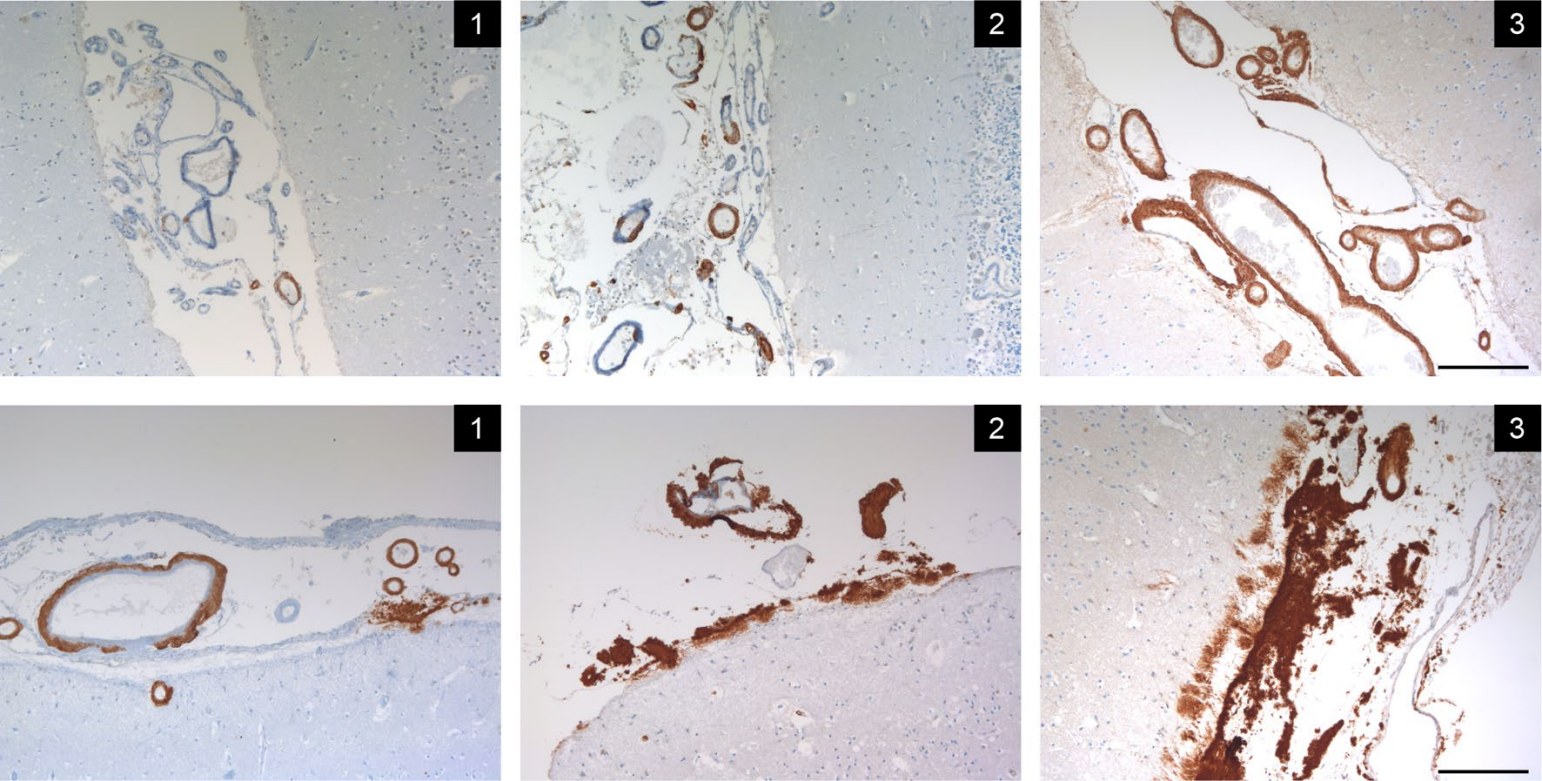
Representative examples of TTR vascular (upper row) and leptomeningeal/subpial deposition (lower row) according to the scale description. TTR immunohistochemistry. Scale bar: 200 µm

Morphological analysis of TTR CNS involvement (leptomeninges, vessels, and parenchyma) was based on anti-TTR immunostained sections. The presence and degree of amyloid deposition was analyzed semi-quantitatively by a neuropathologist (RT) blinded to patient´s clinical features. Amyloid vessel deposition was classified as negative if no TTR positive vessels were found; mild if scattered positivity was found in few vessels; moderate if scattered positivity in many vessels or strong positivity in few vessels was found; and severe if strong positivity in many vessels was found [4] (Fig. 1). In the leptomeninges and brain parenchyma, the amyloid deposition was classified as negative, if no amyloid deposition was found, and as mild, moderate, and severe according to the amount of immunoreactivity present [21] (Fig. 1).

### Statistics

Age at death, sex, mutation, age at disease onset, disease duration, cause of death, and clinical data (TTR-FAP disease stage, intracerebral hemorrhage, Ischemic stroke, cognitive complaints, cranial nerve dysfunction, and liver transplant) were collected for the study. Data are presented in percentages for qualitative variables, mean, and range for quantitative variables.

Normality of the variables age at death, disease duration, and amyloid burden (total number of affected areas and sum of the amyloid scores) was tested using Shapiro–Wilk normality test. Pairwise comparison between groups, based on parametric or non-parametric tests, was performed using one-way ANOVA and Kruskal–Wallis, depending upon the assumptions required. Post hoc testing was performed to identify the groups between which significant differences existed (Dunn–Bonferroni). Pearson´s correlation coefficient test was used to assess correlation between disease duration and number of brain regions with TTR deposition or sum of TTR scores.

The publicly available tool Morpheus (https://software.broadinstitute.org/morpheus) was used to perform unbiased hierarchical clustering. Due to missing data, cervical cord was excluded for this analysis.

Rating data were entered into an Excel spreadsheet and analyzed using Statistical Package for Social Sciences (SPSS) software (version 26.0), Armonk, NY. A *P* value of < 0.05 was considered statistically significant.

## Results

Demographic and clinical data of the 16 cases included in the study are summarized in Table 1. All cases had V30M TTR mutation, except for case #5 with a Ser52Pro mutation. Case #8 was homozygous for V30M TTR mutation. There were 9 females (56%). Patients had a mean age of disease onset of 36.8 years (range: 24–52), a mean age at death of 47.6 years (range: 28–69), and mean disease duration of 10.9 years (range: 3–29). The earliest and predominant manifestation of ATTRv was neuropathy in all cases. The three older patients and with longest disease duration developed cognitive complaints, one of them after cerebral hemorrhage. Cranial nerve disfunction was registered in six patients; some of them with relatively short disease duration. Two patients developed clinically evident hemorrhagic stroke and one developed a lacunar cerebral infarct. Neuropathological details of the 16 cases studied are summarized in the supplementary table 1.

**Table 1:** Patients’ characteristics

Case	#1	#2	#3	#4	#5	#6	#7	#8	#9	#10	#11	#12	#13	#14	#15	#16
Age at death	48	46	37	50	38	36	28	54	48	36	43	41	56	69	68	63
Sex	M	M	M	F	M	F	M	F	M	F	M	F	F	F	F	F
Mutation	–	–	–	V30M	S52P	V30M	V30M	V30M^*^	V30M	V30M	V30M	V30M	V30M	V30M	V30M	V30M
Age at disease onset	41	33	26	39	30	30	24	48	43	30	31	39	37	52	52	34
Disease duration	7	13	13	11	8	6	4	6	5	6	12	3	19	17	16	29
Cause of death	Liver failure	Cardiac arrest	Sepsis	Cardiac arrest	Sepsis	Sepsis	Sepsis	Liver failure	Liver failure	Liver failure	Sepsis	Liver failure	Cachexia	Sepsis	Pneumonia	Renal failure
TTR-FAP disease stage	3	na	3	3	2	1	na	2	2	1	1	1	3	1	3	3
Intracerebral hemorrhage	–	–	–	–	–	–	–	–	–	–	–	–	–	pons	lobar + pons	–
Ischemic stroke	–	LACI	–	–	–	–	–	–	–	–	–	–	–	–	–	–
Cognitive complaints	–	–	–	–	–	–	–	–	–	–	–	–	–	yes	yes	yes
Cranial nerve dysfunction	XII	–	VII, XII	XII	XII	–	–	–	–	–	–	–	VII	VII	–	–
Liver transplant	0	0	0	0	1	1	1	1	1	1	1	1	0	1	1	1

VII: difacial palsy, face fasciculations

XII: tongue fasciculations and/or tongue atrophy

FAP disease stage: 1: symptomatic disease, walks unassisted; 2: walks with assistance; 3: limited to wheelchair

LACI: Lacunar infarct; in this case (#2) a motor lacunar syndrome (isolated hemiplegia with ipsilateral central facial palsy)

*Homozygous mutation

### Morphological characteristics

**Fig. 2:**
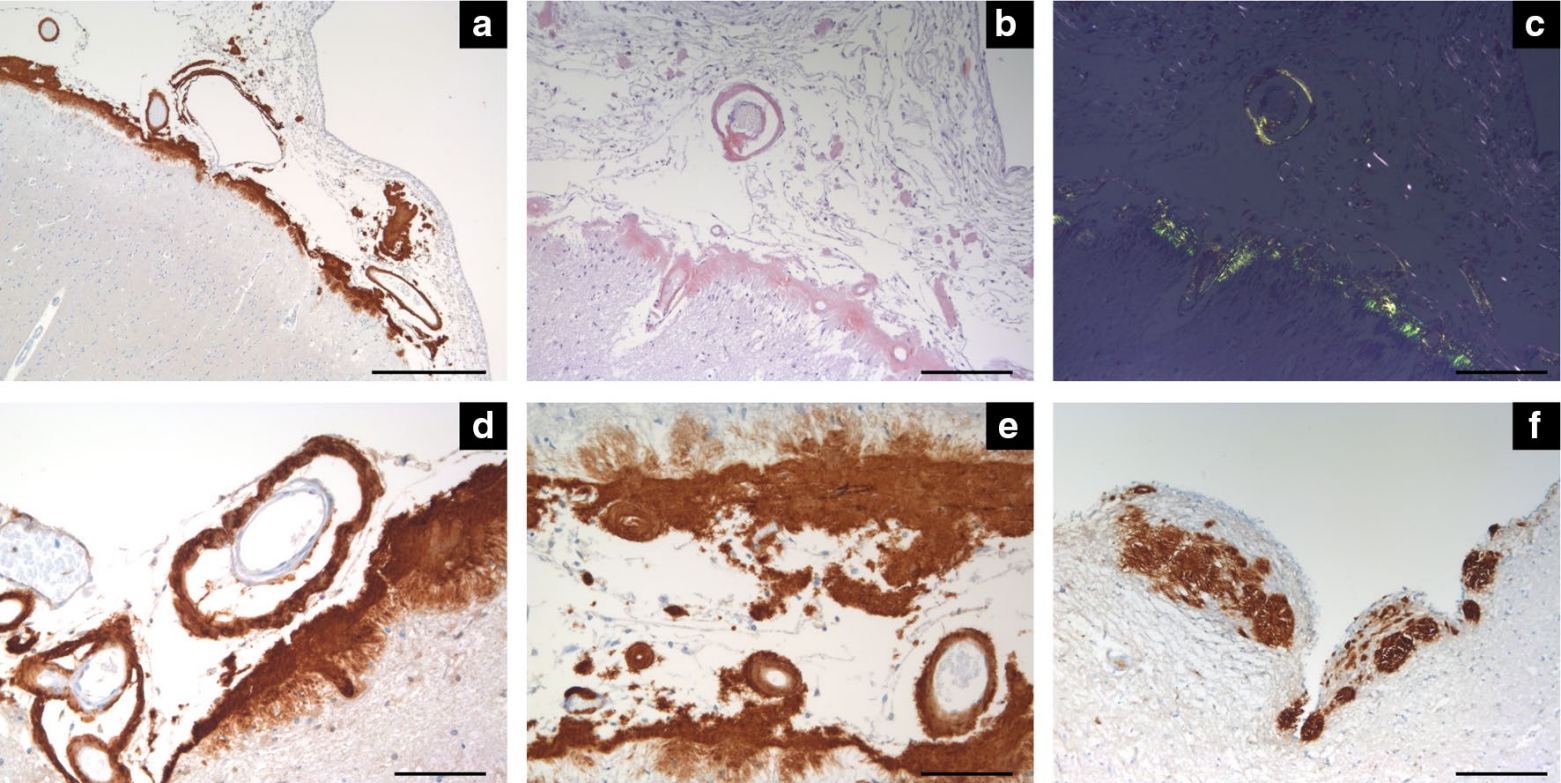
Neuropathology of CNS TTR amyloidosis. Severe leptomeningeal and subpial TTR amyloid deposition associated with severe cerebral amyloid angiopathy with splitting of the vessel wall and double-barreling appearance in several vessels (**a**–**e**). The amyloid deposition has the characteristic green birefringence under polarized light in the Congo red staining (**b** and **c**). Despite massive amyloid deposition, this was restricted to the meningocortical penetrating vessels, with no parenchymal amyloid deposition. In **f**, subependymal amyloid deposition in the fourth ventricle. TTR immunohistochemistry: **a**, **d**–**f** Congo red staining: b and c. Scale bars—**a**: 500 µm; **b**, **c**, **f**: 200 µm; **d** and **e**: 100 µm

Similar to previous descriptions, in the most severely affected cases, there was a severe vasculopathy associated with the CAA, with many vessels showing “double barrel” appearance. The cortical deposition was restricted to the meningocortical penetrating vessels, with no parenchymal amyloid deposits, either in cortex or white matter. Fibrinoid necrosis or capillary CAA was not found in any case. The subependymal deposition, seen in the severely affected cases, was apparently more frequent in the third and fourth ventricle and in the basal ganglia region of the lateral ventricles compared to the temporal horn (Fig. 2).

The case with the longest disease duration (case #16, age 63 at death; 29 years of disease) showed several cortical cerebral and cerebellum microinfarcts of varying age (Supp Fig. 1). Most of these cortical microinfarcts were localized to the superficial layers. The case #15 (age 68; 16 years of disease) showed a temporal glial scarring from a previous lobar hemorrhage. This case also revealed basal ganglia and brainstem old micro-hemorrhagic lesions, and a basal ganglia microinfarct. Case #14 (age 69; 17 years of disease duration) showed an extensive intraparenchymal pons hemorrhage, which was the cause of death, without evidence of other vascular lesions or amyloid deposition in the intraparenchymal vessels.

The subpial TTR amyloid deposits were associated with astrocytosis. In the brainstem and spinal cord, the meningeal and subpial amyloid deposition were frequently in close contact with cranial and spinal nerves but only rarely intranerve deposits were seen (Supp Fig. 1). Mild superficial cortical siderosis was found in two cases (cases #8 and #16; Supp Fig. 1). Additionally, in some cases, there was scattered pigment deposition and hemosiderophages in the leptomeninges and rare hemosiderophages near some capillaries in the cortical white matter.

### TTR topographic distribution according to disease duration

**Fig. 3:**
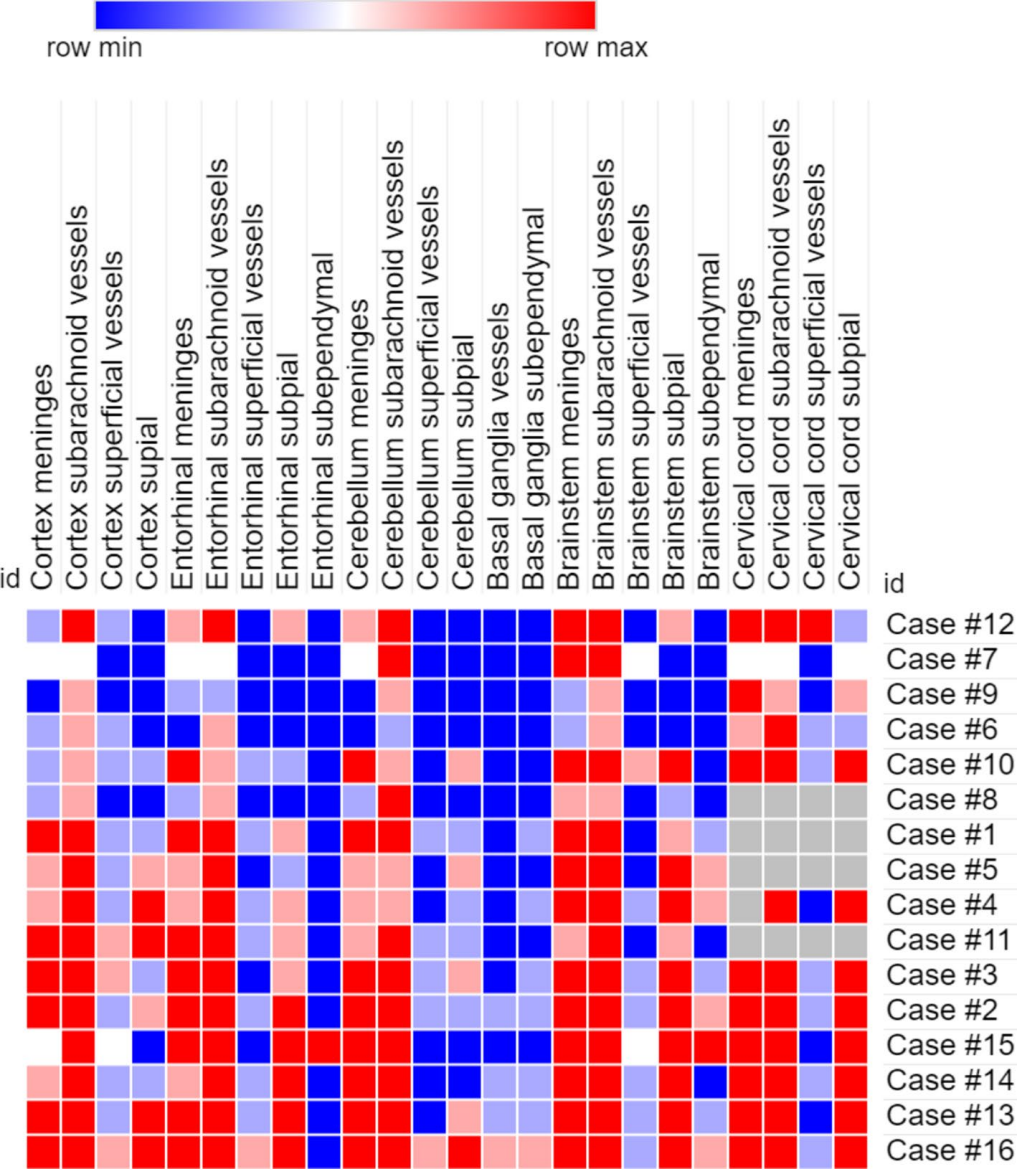
Distribution map of TTR semi-quantitative scores (0–3) in CNS of ATTRv patients. Grey boxes indicate that that anatomical region was not examined

The severity and topographic distribution of TTR amyloid deposition are represented sequentially according to disease duration in Table 2 and in the heat map of Fig. 3.

**Table 2:**
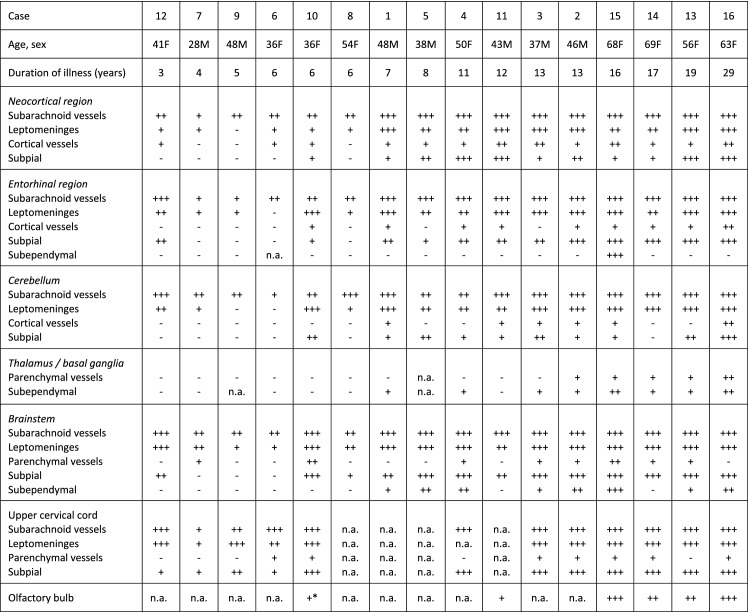
CNS TTR amyloid deposition severity and topographic distribution

*Only present in subarachnoid meningeal vessels

The TTR amyloid deposition, represented as the total number of affected areas or the sum of the scores of the different areas affected, strongly correlated with disease duration, even after controlling for age at death (Supplementary Fig. 2).

**Fig. 4:**
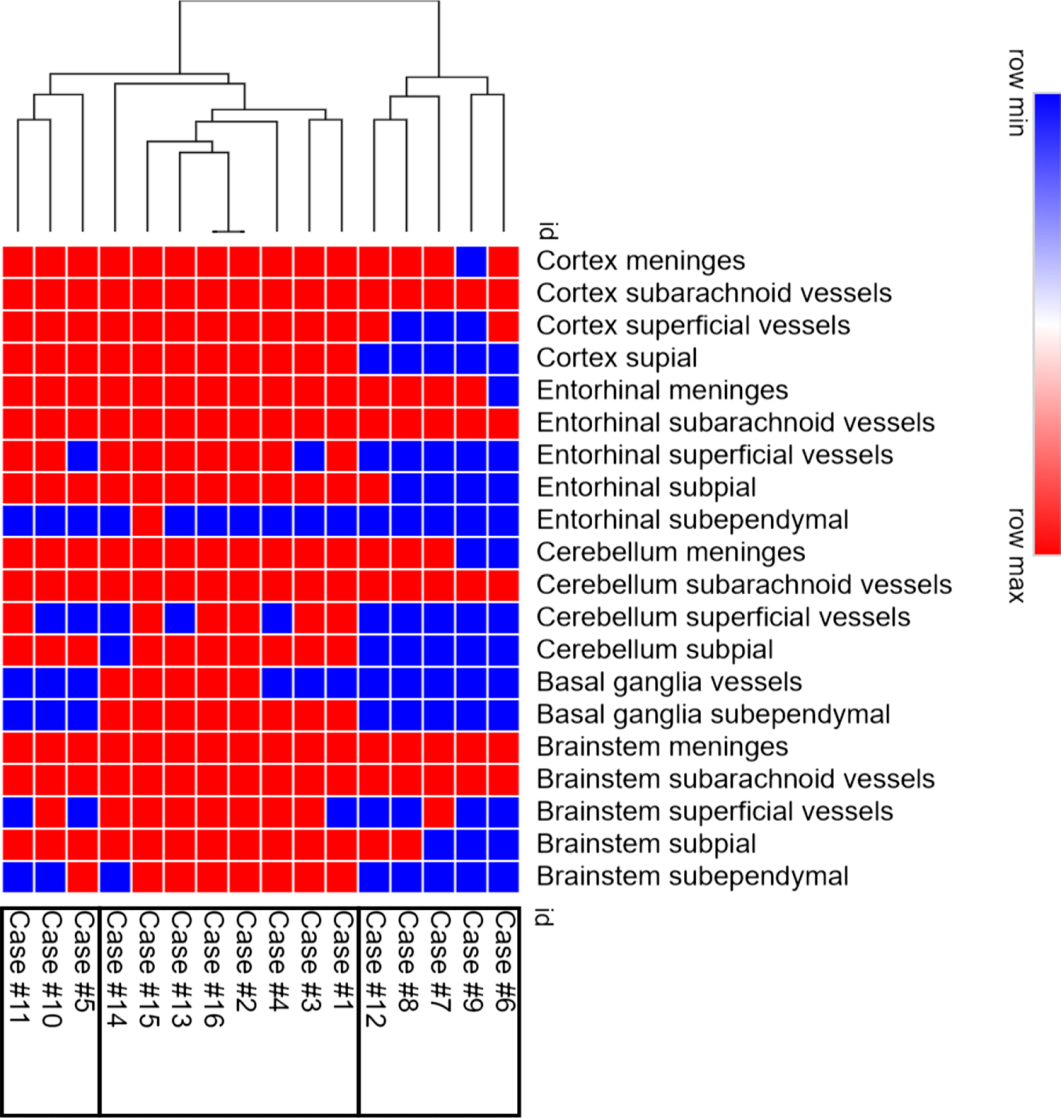
Unsupervised hierarchical clustering according to the affected areas with TTR amyloid deposition. Red boxes represent presence of TTR amyloid deposition and blue boxes represent absence of TTR amyloid deposition

Hierarchical cluster analysis using the binary information amyloid present/non-present identified three different groups (Fig. 4). The groups did not differ regarding sex and age of onset (Chi-square test and one-way ANOVA). The groups differ regarding age (*p* < 0.05) at death and disease duration (*p* < 0.01). Pairwise comparison shows statistical differences only between group 1 and group 3 regarding disease duration (group 3 higher than group 1).

**Fig. 5:**
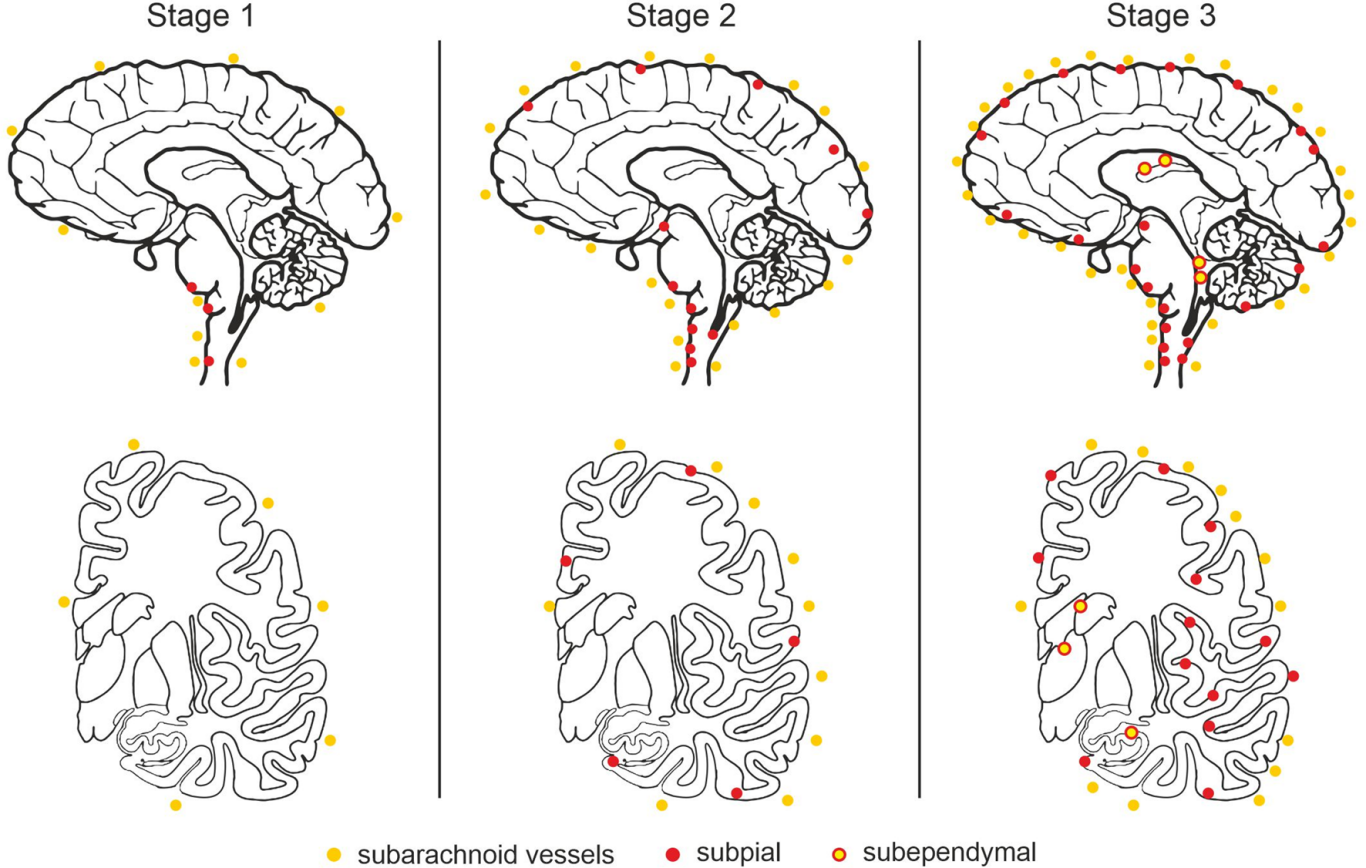
Schematic representation of stages in the distribution of TTR amyloid deposition in a medial view (upper figure) and in a coronal section at level of the basal ganglia of the human brain (lower figure). The amyloid deposition is represented by the colored dots

**Fig. 6:**
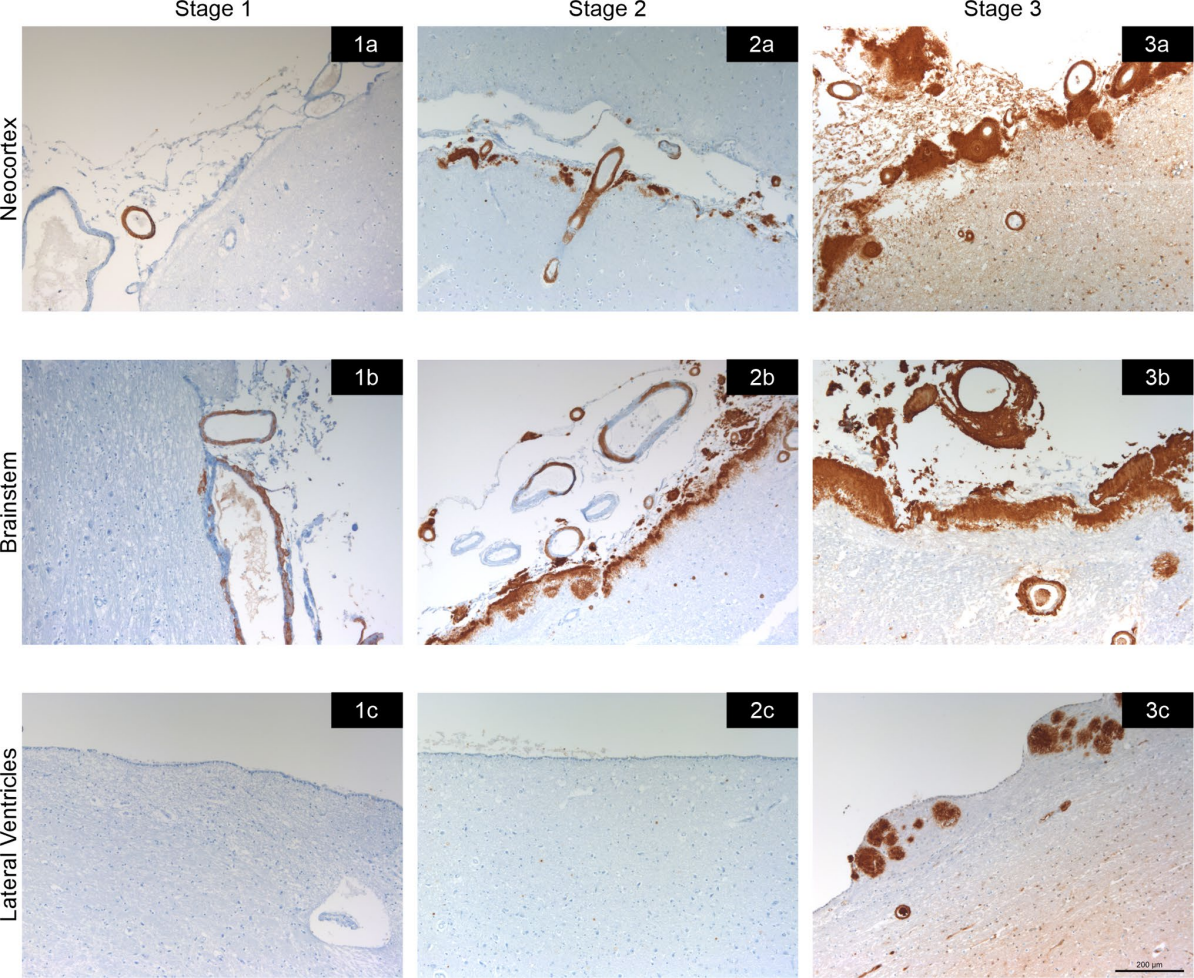
Examples of CNS TTR stages. Stage 1 (1a-1c), stage 2 (2a-2c), and stage 3 (3a-3c). Leptomeninges and subarachnoid vessels are involved earlier, including in the brainstem region in stage 1. In stage 2, there is increased severity of the amyloid deposition and involvement of the perforating cortical vessels and subpial region. Finally, in stage 3, amyloid load continues to increase and there is additional amyloid deposition in the subependymal and basal ganglia vessels near the ependymal lining. Scale bar—200 µm

The anatomical distribution of TTR amyloid deposition in these three groups allowed the determination of 3 stages (Table 3, Figs. 5 and 6). In stage 1, TTR amyloid deposition is restricted to the leptomeninges and subarachnoid vessels, but may be already present in subpial position in brainstem and spinal cord. Very rare superficial cortical vessels can be seen with TTR immunoreactivity. In stage 2, we see amyloid deposition in subpial cortical regions and more frequently in superficial perforating cortical vessels. In stage 3, beyond the affected regions in stage 2 and increasing severity, there is amyloid in subependymal regions and basal ganglia vessels near the ependymal lining. There was a trend towards a higher load of TTR amyloid deposition in cerebellar than cerebral leptomeninges. In the entorhinal subpial region, the amyloid deposition was particularly severe in the ambiens gyrus and subiculum region.

**Table 3:**
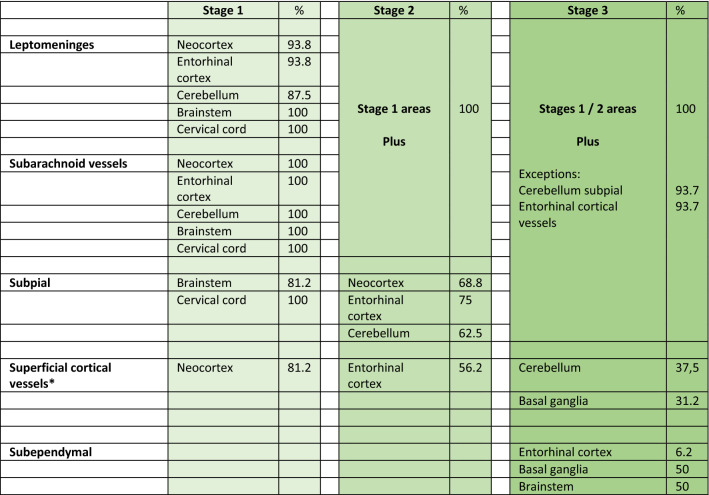
Distribution of TTR amyloid deposition

The percentage was calculated according to the number of cases with amyloid deposition in each region (binary information—present/absent)

*Basal ganglia refers to subependymal vessels

**Fig. 7:**
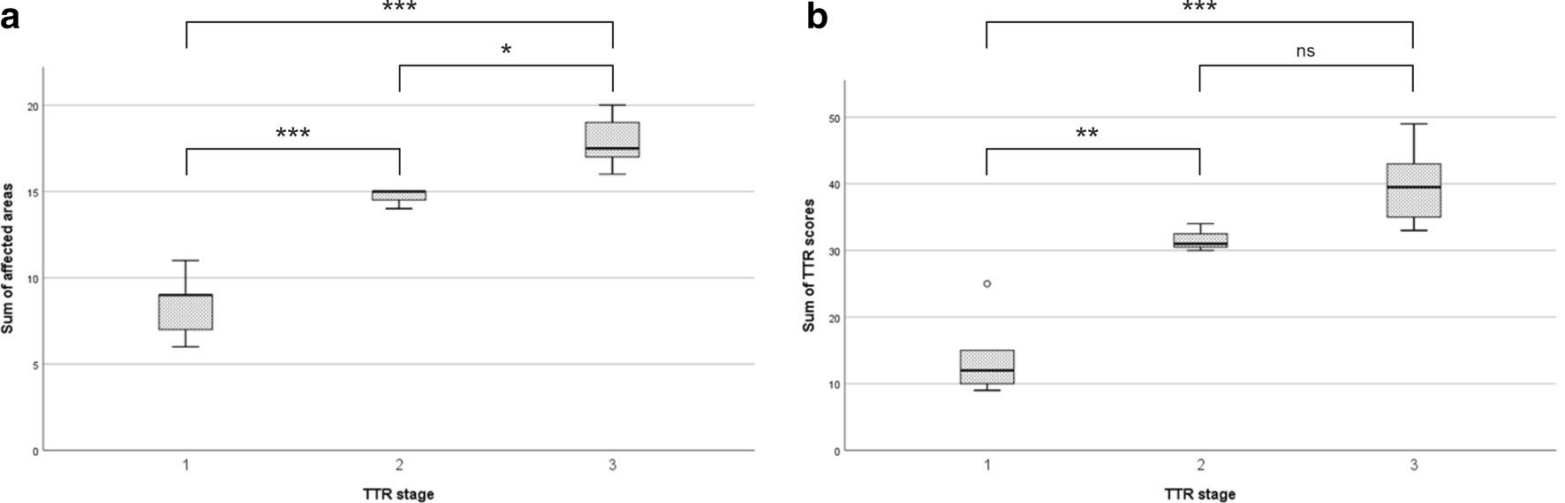
Number affected areas (**a**) and sum of TTR amyloid scores (**b**) according to TTR proposed stages. One-way ANOVA, *p* < 0.0001. *p < 0.05; **p < 0.001; ***p < 0.0001 (Bonferroni correction for multiple tests)

Despite the small number of cases, the validity of these TTR stages was confirmed by demonstrating that the number of affected TTR areas increased with the proposed stage of TTR. Similar results were obtained when considering the sum of the semi-quantitative scale used for each case (Fig. 7).

Frequency and severity of TTR amyloidosis distribution in different lobes were not different, but only few cases and in advanced disease stage were available.

Six cases had olfactory bulb available for analysis (cases #10, #11, #13–16; disease duration from 6 to 29 years), and in all of them, there was amyloid deposition (Supp Fig. 3). The deposition was seen at the nerve periphery and vessels, but in four cases, there was additional intraparenchymal amyloid deposition. In the case with the shortest disease duration (case #10, 6 years of disease duration), amyloid deposition at this level was only detected in the superficial vessels. Brain dura mater was available from three cases, all with long-disease duration (cases #14, #15 and #16), showing severe CAA and frequent amyloid deposits in the fibrous tissue.

### Other neurodegenerative pathologies

The oldest patients (cases #13–16, ages 56–69) showed circumscribed tau pathology in the transenthorhinal region and the locus coeruleus, where neurofibrillary tangles, pre-tangles, and threads were seen (Braak stage I). In these cases, we found tau immunoreactive pathology (AT8ir) in the layer subjacent to the subpial TTR amyloid deposits in the entorhinal cortex and near the hippocampal formation. This finding was absent or very faint in other neocortical areas and absent in brainstem, besides the presence of massive subpial amyloid deposition in these regions. There was no α-synuclein or TDP-43 pathology in any case. Case #16 showed discrete Aβ CAA (Vonsattel grade 1) and discrete parenchymal deposition (Thal phase 2). In this case, there was Aβ immunoreactivity in the subpial deposits, much less prominent compared to TTR.

## Discussion

In this study, we show that the extension of CNS TTR amyloidosis increases with disease duration and that this deposition appears to affect different brain areas in a distinct hierarchical sequence that can be described in 3 stages. The deposition of TTR in the CNS was first evident in the leptomeninges and subarachnoid meningeal vessels, particularly severe in the brainstem and spinal cord, where subpial deposition was already seen in a patient with 3 years of disease duration. In stage 2, the amyloid deposition expanded to subpial cortical regions and was more frequent in the perforating cortical vessels. Finally, in stage 3, it involved subependymal regions and basal ganglia vessels near the ependymal lining. TTR CAA was not accompanied by amyloid deposition in the parenchyma outside the subpial and subependymal regions (either cerebrum, cerebellum, brainstem, or spinal cord), unlike what is seen in the more common sporadic Aβ and some rare forms of familial CAAs, including hereditary cerebral hemorrhage with amyloidosis of Icelandic type (HCHWA-I), familial British dementia (FBD), and familial Danish dementia (FDD) CAA [25]. Additionally, CAA outside the leptomeningeal vessels seemed to be restricted to the most superficial segments of the penetrating cortical vessels. This is consistent with previous pathological descriptions in ATTR [21, 32]. There is one autopsy case description of a V30M ATTR patient who died with intracerebral hemorrhages after 10 years of disease duration in which diffuse intracortical amyloid angiopathy was noted in the cortex [26]. We cannot exclude that additional patient characteristics, including genetically driven, can modulate the pathological phenotype. Interestingly, similarly to the description in Aβ CAA, we found that the penetrating intracortical vessels affected in TTR CAA were less frequent in the entorhinal region and the cerebellum [3, 31]. In the same way, leptomeningeal vessels were affected more frequently and severely than were the cortical ones [4].

It was not possible to assess the differences between frequency and severity of CAA in the different lobes due to the small number of cases with all the regions for study. Amyloid deposition was already severe in some regions in a patient with only 3 years of disease duration, suggesting that CNS amyloid deposition starts in pre-symptomatic phases of the disease. Autopsy studies in patients with very short disease duration or pre-symptomatic phases would be useful to confirm this finding. Additionally, PET imaging, which can mark TTR amyloidosis, can help address this issue.

It is well established that Aβ CAA can result in lobar intracerebral hemorrhage and lobar cerebral microbleeds as well as ischemic lesions, including cortical cerebral microinfarcts and white matter hyperintensities [24]. In this series, only two cases had hemorrhagic lesions and one case had multiple cortical microinfarcts. Single microinfarcts (either cortical or basal ganglia) were seen in additional two cases. There were no evident cortical microbleeds and only two cases showed relatively mild superficial siderosis, in agreement to what was reported in a previous study [21]. The cases with significant hemorrhagic lesions had long-disease duration and old age at onset of the disease (case #14, age 69 at death; 17 years of disease; case #15, age 68 at death, 16 years of disease). Furthermore, some of the vascular lesions at the level of basal ganglia and brainstem were distant from the vessels involved by the amyloid deposition. It is possible that concomitant small vessel disease in these patients contributed or caused these deep brain hemorrhages. This finding reinforces the probable cumulative effect of vascular risk factors and age in the hemorrhagic risk of these patients. Our findings suggest that vascular lesions in V30M ATTRv patients are infrequent and a relatively late feature, being found in this series after at least 12 years of disease duration. On the other hand, case #16, with 29 years of disease duration, had no evidence hemorrhagic lesions, despite showing massive leptomeningeal vessels’ amyloid deposition and extensive cortical microinfarcts.

The scarcity of hemorrhagic lesions in this series suggests that cortical microbleeds are not the primary mechanism involved in TFNEs, the most reported CNS symptom in V30M ATTRv [30]. This is consistent with a previous finding of normal T2* sequences in the brain MRIs of 5 patients with TFNEs [27]. Nevertheless, none of our patients had records of previous TNFEs, so it was not possible to compare this symptom to pathological findings.

In Aβ CAA, neuroimaging studies showed that cortical superficial siderosis (cSS) is one of the most specific and clinically important biomarkers of CAA [7, 23]. cSS is associated with TFNEs and seems to be a risk factor for future symptomatic lobar intracerebral hemorrhage (ICH) [9, 18]. Neuropathologically, it associated with more severe leptomeningeal CAA, compared to parenchymal CAA [20]. In this study, we show that ATTRv has an identical pattern of preferential involvement of leptomeningeal vessels, but we did not find significant cSS. However, the pathological analysis is based on samples of small areas of the brain and might not be fully representative of a patchy process. In a recent neuropathological study on Aβ CAA, areas sampled for histological analysis with MRI-defined cSS showed that these areas corresponded histopathologically to iron-positive haemosiderin deposits in the subarachnoid space and superficial cortical layers, which is indicative of chronic bleeding events originating from the leptomeningeal vessels [10]. In ATTRv, prospective MRI studies are still missing. The pathophysiology of TFNEs remains poorly understood. Seizures or cortical spreading depression could explain the progressive, self-limited symptoms of cortical dysfunction. Cortical spreading depression or depolarization is a phenomenon of sequential depolarization of contiguous cortical cells that results in a transient loss of function. It underlies migraine auras, but it can be induced by different cortical insults. In Aβ CAA, it seems possible that subarachnoid hemorrhage or superficial siderosis is the direct or indirect triggers of cortical spreading depression [29]. In ATTRv, the severity of subpial amyloid deposition found in this series raises the possibility of a direct role of the amyloid aggregates in the pathological changes found in the adjacent cortex.

Despite the retrospective nature of the study, we were able to identify clinical records of cranial nerve dysfunction (facial and hypoglossal nerves) in 6 patients. Signs of brainstem or cranial nerve dysfunction have been sporadically mentioned in ATTRv. Recent studies showed higher prevalence of sensorineural hearing loss in ATTRv patients than the general population [6], and dysphonia and dysphagia seem frequent in late onset ATTRv patients [5]. The early and severe subpial brainstem amyloid deposition found in this study supports a direct effect of amyloid on the cranial nerves at their emergence from the brainstem. The amyloid deposition found on the olfactory bulbs also suggests that olfactory dysfunction may be a CNS symptom in V30M ATTRv and warrants further investigation. In fact, there is a lack of studies on ATTRv that systematically evaluate the CNS symptoms and their prognostic implications.

Regarding neurodegenerative associated pathology, abnormal tau immunoreactive neurites have been described in relation to CAA or subpial regions in others amyloidosis [19]. In ATTR, a previous study reported a case of a 72-year-old male with the rare Tyr69His TTR gene variant, dementia, and ataxia [34]. The authors found phospho-tau aggregates subjacent to the subpial TTR amyloid deposits in all regions of the neocortex, including the primary motor and striate cortices, and suggest a potential link between TTR amyloid and neocortical tauopathy. In our series, phosfo-tau was only present in the entorhinal region despite similar amounts of subpial amyloid deposits in all neocortical regions. The cortical tau pathology was also circumscribed to the transenthorrinal cortex, indicative of Braak stage I and in locus coeruleus, similar to what is usually found in clinicopathological studies in young and middle-aged cognitive unimpaired subjects [8, 15]. At least in Val30M ATTRv, it seems that abnormal tau pathology near subpial amyloid deposits follows the typical topographical progression described by Braak [8]. Nevertheless, due to the increasing longevity of ATTRv patients, it will be important to understand the effect of age on TTR pathology, particularly the age-related neurodegenerative pathology.

This study has several limitations, namely the number of patients studied and the fact that the majority had a long-disease duration prior to death. The retrospective nature of the study can be misleading regarding the assumed absence of CNS clinical symptoms. Nevertheless, the available neuropathological descriptions of the disease rely mostly on case reports or smaller cohorts [32]. We decided to include in the analysis the case with a Ser52Pro mutation, because the natural history of this mutation is similar to the V30M ATTRv [7]. The neuropathological findings of this case were similar to the V30M TTR cases. The case carrying the homozygous V30M TTR mutation did not have distinct clinical or pathological features compared to the heterozygous cases. A recent V30M cohort study in Sweden showed that homozygous V30M patients had similar disease expression and severity to their heterozygous counterparts [17].

## Conclusion

Our results suggest that CNS pathological involvement in V30M ATTRv occurs early in the disease course, probably starting in pre-symptomatic phases. The progression follows a hierarchical sequence, with brainstem severely affected in earlier stages. Vascular lesions or superficial cortical siderosis are not frequent features of the disease. The clinical correlates of the pathological findings remain to be explored. Future studies with more patients from different populations and TTR mutations will be important to confirm these findings, eventually extending in detail the neuropathological signatures of ATTR amyloidosis in the CNS.

Defining stages of TTR pathology helps to identify preclinical or early stage cases for the better understanding of early pathogenic events and can be informative to understand neuroimaging biomarkers.

## Supplementary Information

Below is the link to the electronic supplementary material.

Supplementary Figure 1. Neuropathology of CNS TTR amyloidosis. Example of the multiple cortical microinfarcts found in case #16 (a). Amyloid deposition surrounding nerve roots of cervical spinal cord (b, case #10). Rarely, intra-fascicular nerve deposition was found, as depicted in the trigeminal nerve near the pons in c (case #15). Mild superficial cortical siderosis in cases #8 (d and e, cerebellum and cortex respectively) and #16 (f, cortex). H&E: a; TTR immunohistochemistry: b and c; Perls staining: d – f. Scale bars - A: 1mm; B and C: 500 µm; D - F: 100 µm. (TIF 8395 kb)

Supplementary Figure 2. Correlation between TTR amyloid deposition and disease duration. a – Correlation between the sum of analyzed areas with TTR deposition (semi-quantitative score ≥ 1) and disease duration (Pearson´s correlation 0.713 and 0.599 when controlling for age at death, p < 0.01 and p < 0.05, respectively); b – Correlation between the sum of the total scores of TTR amyloid deposition and disease duration (Pearson´s correlation 0.778 and 0.684 when controlling for age at death, p < 0.001 and p < 0.01, respectively) (TIF 5599 kb)

Supplementary Figure 3. Olfactory bulb. a - TTR amyloid deposition in the periphery of the nerve and vessels (case #11). b - TTR deposition restricted to the subarachnoid vicinity vessels (case #10). Severe amyloid deposition in the olfactory bulb in case #13 (c) compared to their absence in case #10 (d). Scale bar - 200 µm (TIF 5686 kb)

Supplementary file4 (DOCX 21 kb)
