# Supplementary figures and images for: Neuropathology of central nervous system involvement in TTR amyloidosis
Source: Acta Neuropathol. 2022 Oct 6;145(1):113–26. doi: 10.1007/s00401-022-02501-9 (PMC9807485; doi:10.1007/s00401-022-02501-9)

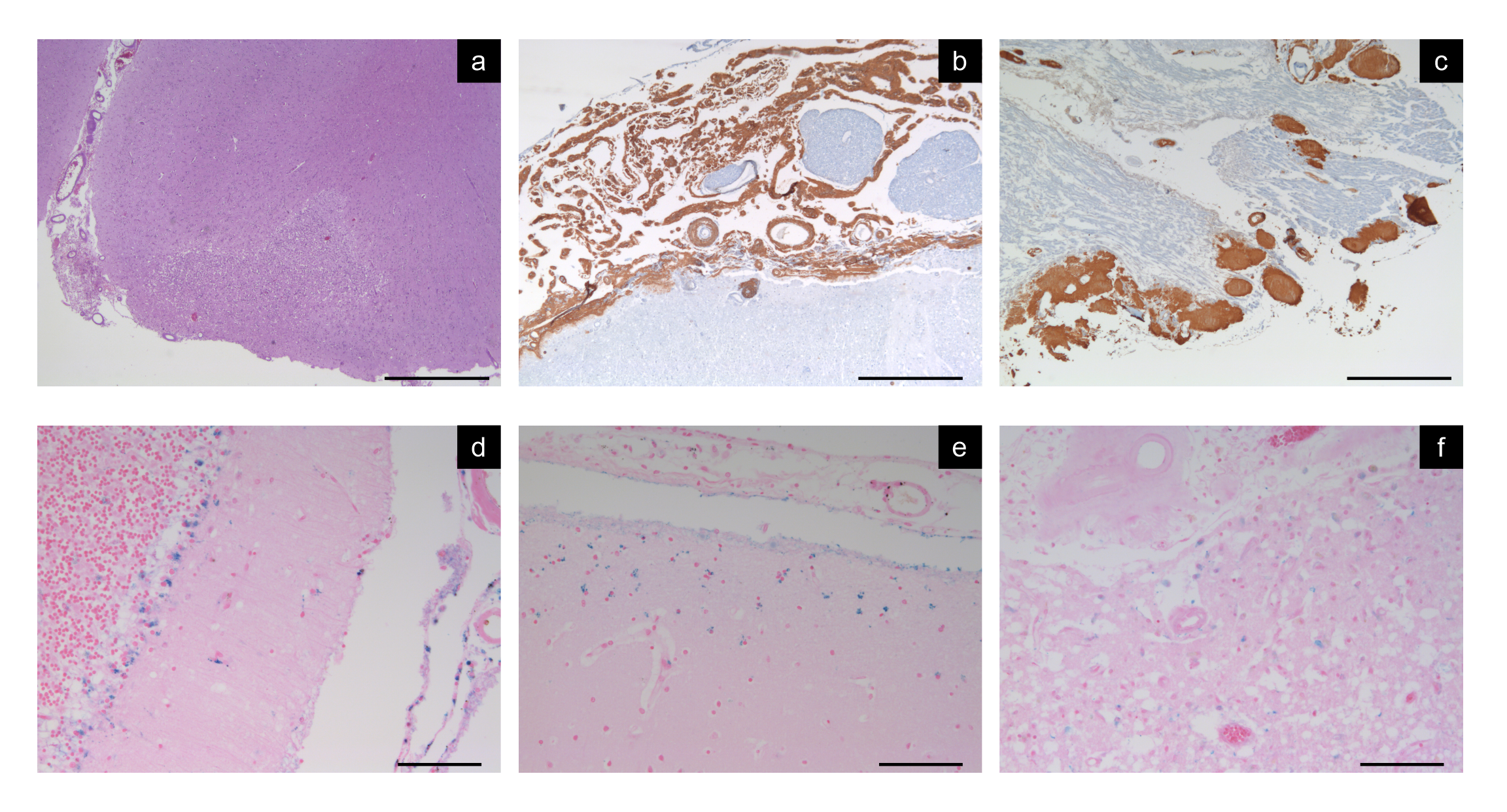

Supplement: Supplementary file 1 — Supplementary Figure 1. Neuropathology of CNS TTR amyloidosis. Example of the multiple cortical microinfarcts found in case #16 (a). Amyloid deposition surrounding nerve roots of cervical spinal cord (b, case #10). Rarely, intra-fascicular nerve deposition was found, as depicted in the trigeminal nerve near the pons in c (case #15). Mild superficial cortical siderosis in cases #8 (d and e, cerebellum and cortex respectively) and #16 (f, cortex). H&E: a; TTR immunohistochemistry: b and c; Perls staining: d – f. Scale bars - A: 1mm; B and C: 500 µm; D - F: 100 µm. (TIF 8395 kb) [file 401_2022_2501_MOESM1_ESM.tif]

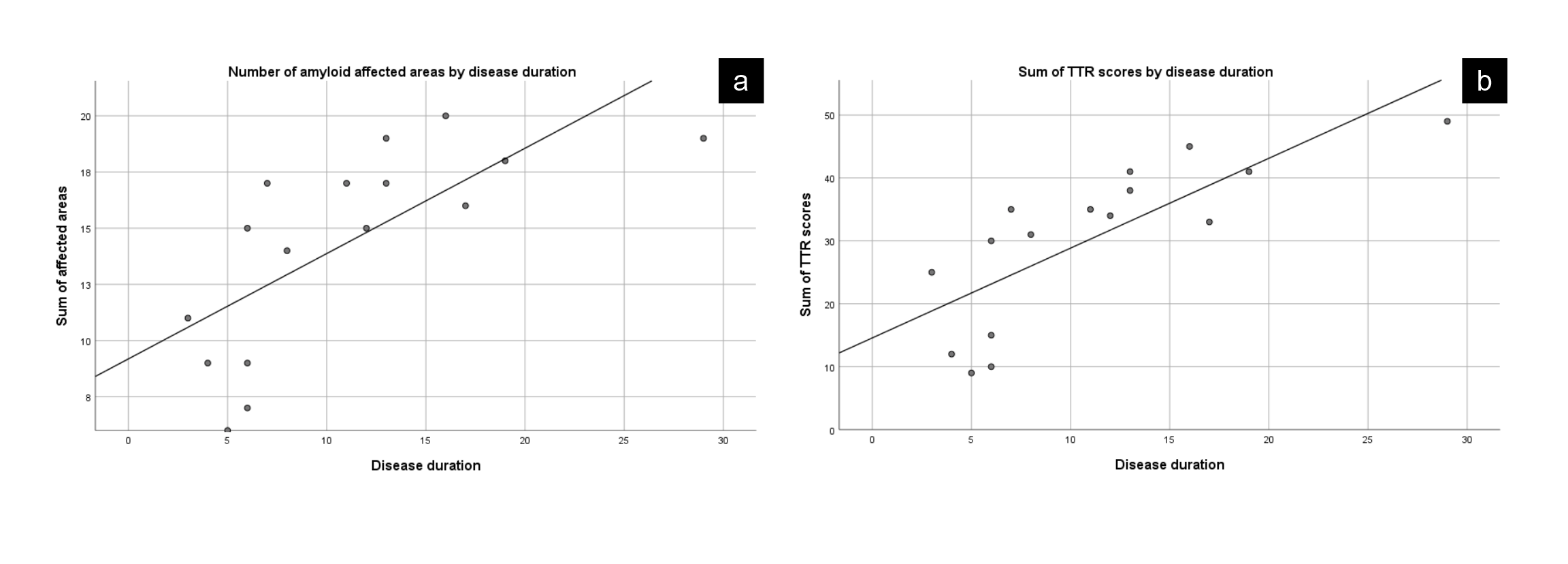

Supplement: Supplementary file 2 — Supplementary Figure 2. Correlation between TTR amyloid deposition and disease duration. a – Correlation between the sum of analyzed areas with TTR deposition (semi-quantitative score ≥ 1) and disease duration (Pearson´s correlation 0.713 and 0.599 when controlling for age at death, p < 0.01 and p < 0.05, respectively); b – Correlation between the sum of the total scores of TTR amyloid deposition and disease duration (Pearson´s correlation 0.778 and 0.684 when controlling for age at death, p < 0.001 and p < 0.01, respectively) (TIF 5599 kb) [file 401_2022_2501_MOESM2_ESM.tif]

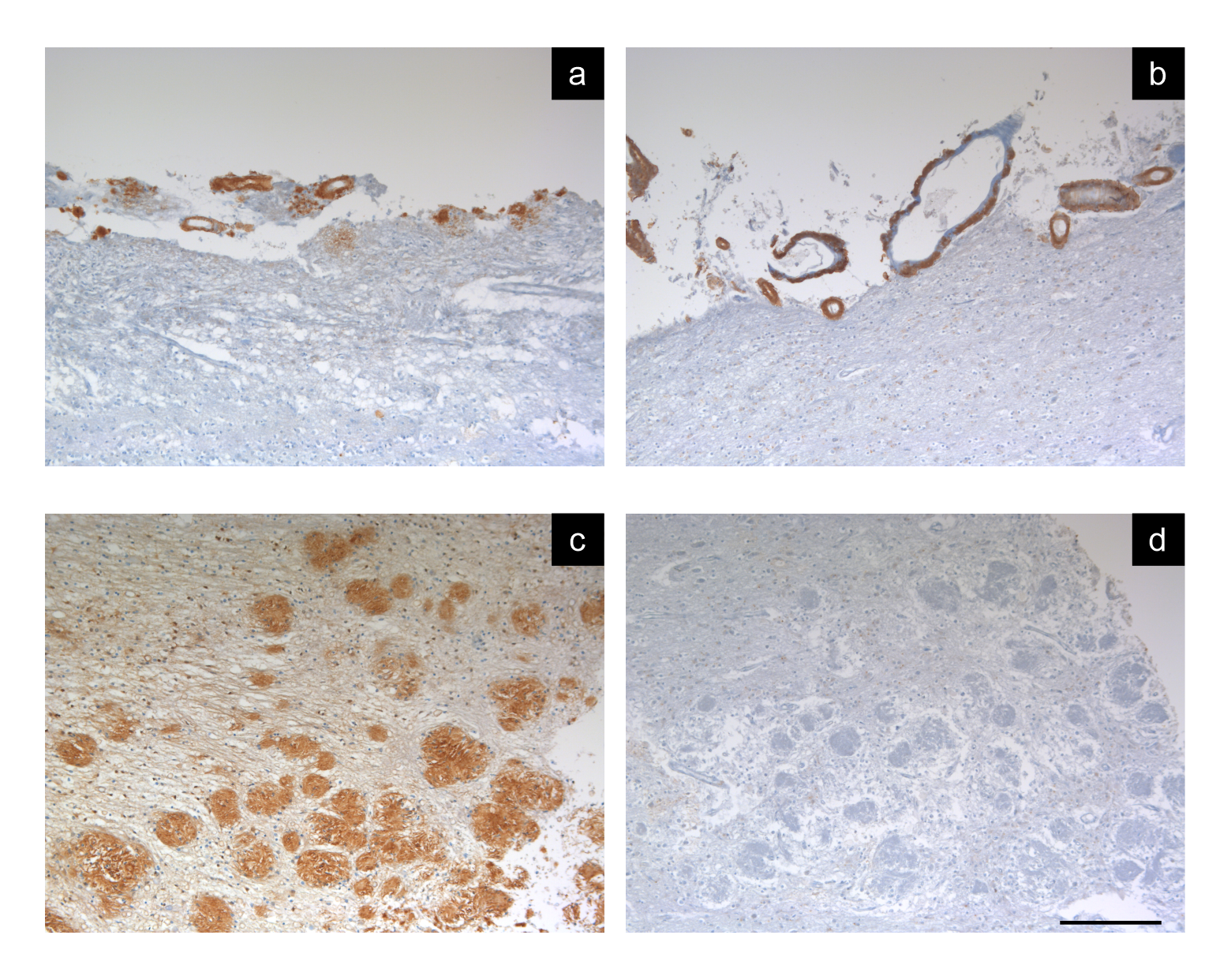

Supplement: Supplementary file 3 — Supplementary Figure 3. Olfactory bulb. a - TTR amyloid deposition in the periphery of the nerve and vessels (case #11). b - TTR deposition restricted to the subarachnoid vicinity vessels (case #10). Severe amyloid deposition in the olfactory bulb in case #13 (c) compared to their absence in case #10 (d). Scale bar - 200 µm (TIF 5686 kb) [file 401_2022_2501_MOESM3_ESM.tif]
