# Supplementary material for: Neuropathology of central nervous system involvement in TTR amyloidosis
Source: Acta Neuropathol. 2022 Oct 6;145(1):113–26. doi: 10.1007/s00401-022-02501-9 (PMC9807485; doi:10.1007/s00401-022-02501-9)
Supplement: Supplementary file 4 — Supplementary file4 (DOCX 21 kb) [file 401_2022_2501_MOESM4_ESM.docx]

Table 1: Neuropathological characteristics

| **Case** | **Age** | **Sex** | **CAA TTR** | **CAA Aβ** | **Braak & Braak stage** | **Thal Phase** | **TDP43** | **α-syn** | **SVD** | **CSS** | **Hemorrhagic lesions** | **Ischemic lesions** |
| --- | --- | --- | --- | --- | --- | --- | --- | --- | --- | --- | --- | --- |
| 1 | 48 | M | +++ | 0 | 0 | 0 | - | - | - | - | Absent | Absent |
| 2 | 46 | M | +++ | 0 | 0 | 0 | - | - | - | - | Absent | Absent |
| 3 | 37 | M | +++ | 0 | 0 | 0 | - | - | - | - | Absent | Absent |
| 4 | 50 | F | +++ | 0 | 0 | 0 | - | - | - | - | Absent | Absent |
| 5 | 38 | M | +++ | 0 | 0 | 0 | - | - | - | - | Absent | Absent |
| 6 | 36 | F | +++ | 0 | 0 | 0 | - | - | - | - | Absent | Absent |
| 7 | 29 | M | +++ | 0 | 0 | 0 | - | - | - | - | Absent | Absent |
| 8 | 54 | F | +++ | 0 | 0^+^ | 0 | - | - | + | ++ | Absent | Absent |
| 9 | 48 | M | +++ | 0 | 0^+^ | 0 | - | - | + | - | Absent | Absent |
| 10 | 36 | F | +++ | 0 | 0 | 0 | - | - | - | - | Absent | single basal ganglia microinfarct |
| 11 | 43 | M | +++ | 0 | 0 | 0 | - | - | - | - | Absent | single cortical entorhinal microinfarct |
| 12 | 41 | F | +++ | 0 | 0 | 0 | - | - | - | - | Absent | Absent |
| 13 | 56 | F | +++ | 0^+^ | I | 0 | - | - | ++ | - | Absent | single cortical and basal ganglia microinfarct |
| 14 | 69 | F | +++ | 0 | I | 0 | - | - | + | - | pons | Absent |
| 15 | 68 | F | +++ | 0^+^ | I | 0 | - | - | ++ | - | lobar, basal ganglia and pons | Absent |
| 16 | 63 | F | +++ | + | I | 2 | - | - | ++ | + | Absent | neocortex, cerebellar cortex and brainstem multiple microinfarcts |

Legend: CAA – cerebral amyloid angiopathy; CSS – cortical superficial siderosis; NFT – neurofibrillary tangles; SVD – small vessel disease; syn – synuclein; TDP43 - TAR DNA-binding protein 43; 0^+^ scattered pigment in some leptomeningeal vessels or scattered tau dystrophic neurites/pre-tangles; + mild, ++ moderate, +++ severe (for the characteristics detailed in the column) .
